# Development of 5123 Intron-Length Polymorphic Markers for Large-Scale Genotyping Applications in Foxtail Millet

**DOI:** 10.1093/dnares/dst039

**Published:** 2013-10-01

**Authors:** Mehanathan Muthamilarasan, B. Venkata Suresh, Garima Pandey, Kajal Kumari, Swarup Kumar Parida, Manoj Prasad

**Affiliations:** National Institute of Plant Genome Research (NIPGR), Aruna Asaf Ali Marg, New Delhi 110 067, India

**Keywords:** comparative mapping, foxtail millet (*Setaria italica* L.), intron-length polymorphism (ILP), physical mapping, transferability

## Abstract

Generating genomic resources in terms of molecular markers is imperative in molecular breeding for crop improvement. Though development and application of microsatellite markers in large-scale was reported in the model crop foxtail millet, no such large-scale study was conducted for intron-length polymorphic (ILP) markers. Considering this, we developed 5123 ILP markers, of which 4049 were physically mapped onto 9 chromosomes of foxtail millet. BLAST analysis of 5123 expressed sequence tags (ESTs) suggested the function for ∼71.5% ESTs and grouped them into 5 different functional categories. About 440 selected primer pairs representing the foxtail millet genome and the different functional groups showed high-level of cross-genera amplification at an average of ∼85% in eight millets and five non-millet species. The efficacy of the ILP markers for distinguishing the foxtail millet is demonstrated by observed heterozygosity (0.20) and Nei's average gene diversity (0.22). *In silico* comparative mapping of physically mapped ILP markers demonstrated substantial percentage of sequence-based orthology and syntenic relationship between foxtail millet chromosomes and sorghum (∼50%), maize (∼46%), rice (∼21%) and *Brachypodium* (∼21%) chromosomes. Hence, for the first time, we developed large-scale ILP markers in foxtail millet and demonstrated their utility in germplasm characterization, transferability, phylogenetics and comparative mapping studies in millets and bioenergy grass species.

## 1. Introduction

Foxtail millet [(*Setaria italica* (L.) P. Beauv] has recently been regarded as a tractable model crop due to its small genome (∼ 515 Mb; 2*n* = 2*x* = 18), low amount of repetitive DNA, inbreeding nature and short life cycle.^1,2^ Moreover, its close relatedness to several bioenergy crops with complex genomes such as switchgrass (*Panicum virgatum*), napiergrass (*Pennisetum purpureum*) and pearl millet (*Pennisetum glaucum*) and its potential abiotic stress tolerance adds up to the merits of foxtail millet as an experimental model crop for exploring various plant architectural traits, evolutionary genomics and physiological attributes of the C_4_ Panicoid grass crops.^1–3^ Since foxtail millet has one of the largest sets of both cultivated and wild-type germplasm rich in phenotypic variations, it appears promising for association mapping and allele mining of elite and novel variants to be integrated in crop improvement programmes.^2,4,5^ Hence, considering the importance of foxtail millet, the Joint Genome Institute (JGI) of the Department of Energy, USA, and BGI (formerly Beijing Genome Initiative), China, have recently sequenced its genome.^6,7^ The availability of genomic sequence has motivated the scientific community to generate genomic resources, which ultimately had resulted in the development of large-scale sequence-based genomic^8^ and genic expressed sequence tag (EST)-derived microsatellite markers.^9^ Noteworthy, the recently developed ‘Foxtail millet Marker Database (FmMDb)’ (http://www.nipgr.res.in/foxtail.html)^10^ encompasses all these generated genomic resources for the benefit of research community aiming in genetic improvement of target millet and its related bioenergy crop species, thus bridging the gap between the researchers and breeders.

DNA markers such as restriction fragment-length polymorphism,^11^ random amplified polymorphic DNA,^12^ amplified fragment-length polymorphism,^13^ simple sequence repeat polymorphism (SSR),^14^ single-nucleotide polymorphism^15^ and intron-length polymorphism (ILP)^16^ exploit the variations, or polymorphisms in DNA sequences are used in various genotyping applications in crop plants. Of these, ILP markers are unique since they are gene-specific, co-dominant, hypervariable, neutral, convenient and reliable.^17^ ILP markers utilize the variation in the intron sequences and are the most easily recognizable type as it could be detected by PCR with primers designed on exons flanking the target intron.^18^ Thus, these markers in spite of being derived from gene sequences showed higher intra-specific polymorphism in plant species than other kinds of markers. In addition to being sequence-tagged sites markers,^19^ ILP markers have high transferability rates among related plant species.^18,20^ In order to facilitate straightforward mining of ILP markers, Yang *et al*.^17^ has developed a web-based database platform named PIP (potential intron polymorphism) to provide detailed information of the PIP markers and homologous relationships among PIP markers from different species.

Regardless of these advantages, very few reports are available on development of ILP markers in plant species when compared with reports on other DNA markers. Noteworthy, till now only one study has been carried out on ILP markers in foxtail millet,^20^ where about 98 markers were developed and characterized. Hence, in view of the importance of ILPs and the availability of less significant number of ILP markers in foxtail millet, the present study was conducted aiming at: (i) developing ILP markers at large-scale from the entire set of publicly available foxtail millet ESTs, (ii) demonstrating the applicability of the ILP markers in examining genetic diversity and cross-species transferability and (iii) developing physical map for studying *in silico* ILP marker-based comparative mapping between foxtail millet and other grass species such as sorghum, maize, rice and *Brachypodium*.

## 2. Materials and methods

### 2.1. Plant material and DNA isolation

The details of plant materials used in the study are summarized in Supplementary Table S1. The seeds of all the investigated species were surface-sterilized in 3% sodium hypochlorite for 20 min, rinsed with sterile distilled water and were germinated in greenhouse. The genomic DNA was isolated from the fresh young leaves using the CTAB method as described elsewhere.^21^ The DNA was purified and then quantified on agarose gel by comparison with 50 ng/μl of standard lambda (*λ*) DNA marker (NEB).

### 2.2. Development of putative ILP markers

The publicly available EST sequences of *S. italica* were searched and retrieved from NCBI dbEST (ftp://ftp.ncbi.nih.gov/blast/db/). Approximately 66 027 ESTs were used for the unigene definition using the CD-HIT (Cluster Database at High Identity with Tolerance) software tool (http://weizhong-lab.ucsd.edu/cdhit_suite/cgi-bin/index.cgi) for redundancy minimization and assembling of sequences. The non-redundant ESTs were used for the development of specific intron-based markers. Using rice genomic sequence as reference, PIP database (http://ibi.zju.edu.cn/pgl/pip/)^17^ was used to predict intron positions in the EST sequences and then designed a pair of primers flanking the intron position. A query EST was considered to be homologous to a subject-coding sequence only if there were at least 100 bp overlapping and 80% similarity between them. The corresponding position and length of identified introns from the subject species were obtained from the PIP database.^17^ To cross-check the primer-designing potential of ILP markers and validate the results of the PIP database, the forward and reverse primers designed for the ILP markers were BLAST-searched against latest released foxtail millet pseudomolecules of nine chromosomes (http://www.phytozome.net).

### 2.3. Functional annotation and physical mapping of ILP markers

The putative functions of the ILP markers were assigned by executing BLASTX search of respective marker encompassing EST sequences against the non-redundant database at NCBI (http://blast.ncbi.nlm.nih.gov/Blast.cgi) with default search parameters. The ILP markers were BLAST-searched against the whole-genome sequences of foxtail millet available at Phytozome (http://www.phytozome.net) and plotted individually on each of the nine foxtail millet chromosomes according to their ascending order of physical position (bp), from the short-arm telomere to the long-arm telomere and finally visualized in the MapChart software.^22^ To further validate the BLAST results of *in silico* physical mapping, we re-analysed the forward and reverse primers of ILP markers against foxtail millet chromosome pseudomolecules using the ePCR program (www.ncbi.nlm.nih.gov/sutils/e-pcr).^23^

### 2.4. Validation of ILP markers

The ILPs were amplified in a 25 µl total volume containing 1 unit of Taq DNA polymerase (Sigma), 50 ng of genomic DNA, 10 µmol/l of each primer, 0.5 mmol/l of each dNTPs and 2.5 µl of 10× PCR reaction buffer [500 mM KCl, 200 mM Tris–HCl (pH 8.4) and 3 mM MgCl_2_] in an iCycler thermal controller (Bio-Rad). The PCR profile was an initial denaturation of 3 min at 94°C, followed by 35 cycles of 60 s at 94°C, 60 s at 50–55°C and 2 min at 72°C, and a final extension of 10 min at 72°C. The amplicons were resolved on 2% agarose gel (Cambrex, USA) in Tris-borate EDTA buffer (pH 8.0), stained with ethidium bromide and analysed using GelDoc-It™ imaging system (UVP). The fragment size for each locus was determined by 100 bp standard size markers (NEB). Results were confirmed by three replicate assays.

The amplified products (alleles) from millet and non-millet species were eluted and cloned into pGEM^(R)^-T Easy vector (Promega) following the manufacturer's instructions. The recombinant plasmids were purified using *AccuPrep* Plasmid MiniPrep DNA Extraction Kit (Bioneer) following the manufacturer's protocol. The plasmids were sequenced in an automated sequencer (3730xI DNA Analyzer, Applied Biosystems) using M13 forward and reverse primers. The sequence information was used to construct multiple sequence alignment along with a reference *S. italica* sequence using the ClustalW2 program (http://www.ebi.ac.uk/Tools/clustalw2/index.html).

### 2.5. Genetic diversity

The ILP marker profiles amplified among 96 foxtail millet accessions were scored manually; each allele was scored as present (1) or absent (0) for each of the ILP loci. Polymorphic informative content (PIC) values were calculated according to Roldán-Ruiz *et al*.^24^ as PIC*_i_* = 2*f_i_*(1 − *f_i_*), where *f_i_* is the frequency of the amplified allele (band present), and (1 − *f_i_*) is the frequency of the null allele (band absent) of marker *i.* Using pairwise similarity matrix of Jaccard's coefficient,^25^ the level of genetic diversity among foxtail millet accessions was calculated and a phylogenetic tree was constructed by unweighted pair-group method of arithmetic average, neighbour-joining (NJoin) module of the NTSYS-pc software v2.02.^26^ The genetic relationships among millets and non-millet grass species based on cross-transferability of ILP markers were determined based on Nei (1983)^27^ diversity co-efficient, and a phylogenetic tree was constructed using the neighbour-joining (NJ) tree interface of the PowerMarker software ver2.5.^28^ The observed heterozygosity (*H*_O_), Nei's average gene diversity^27^ and fixation index (*F*_IS_) were also computed using the PowerMarker software ver2.5.^28^ Correlation analysis among PIC and the number of alleles were examined using the GraphPad InStat software v3.10 (www.graphpad.com).

### 2.6. *In silico* comparative genome mapping

The ILP markers that were physically mapped on the nine chromosomes of foxtail millet were BLASTN-searched against genome sequences of sorghum, maize, rice and *Brachypodium* (www.phytozome.net) to develop marker-based syntenic relationships among the chromosomes of foxtail millet and three other grass species. A cut-off bit score of 54.7 and an *E*-value of <1e − 05 were considered optimum for BLASTN analysis. The marker-based syntenic relationships among foxtail millet, sorghum, maize, rice and *Brachypodium* were finally visualized with visualization blocks in the Circos software v0.55 (http://circos.ca).^29^

## 3. Results and discussion

### 3.1. Development of ILP markers and physical mapping in foxtail millet genome

**Figure 1.**
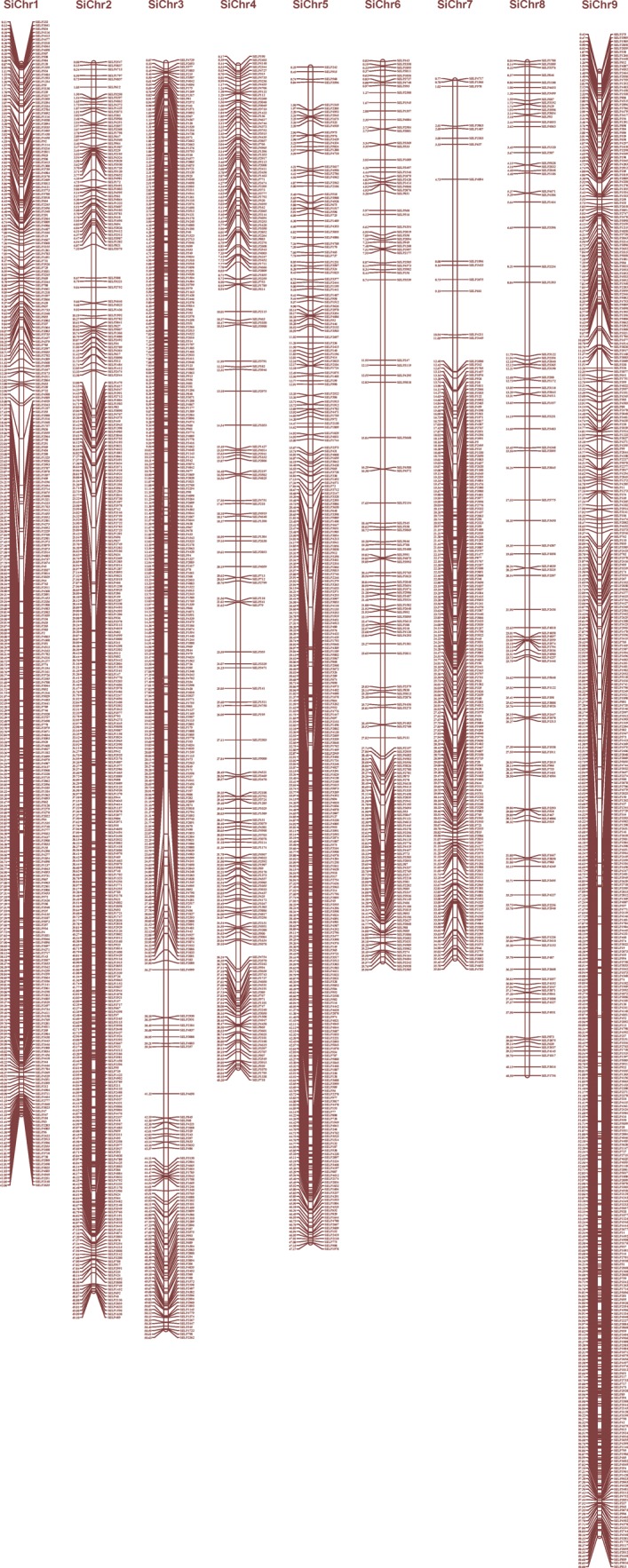
Physical genomic distribution of 4049 ILP markers on the nine chromosomes of foxtail millet genome. The number on the right side of the bar denotes the identity of foxtail millet ILP markers (SiILP, *Setaria italica* intron-length polymorphic marker) and that on the left side indicates the physical position of mapped markers in mega base-pairs (Mb).

A set of 66 027 EST sequences of *S. italica* produced 24 828 non-redundant ESTs, which were used for generating ILP markers by using rice as reference genome in the PIP database.^17^ A total of 5123 ILP markers (20.6%) were generated out of 24 828 EST sequences with an average frequency of ∼12.6 ILP markers per megabase genomic sequences (Supplementary Table S2). BLAST analysis of 5123 ILP markers against the foxtail millet genome showed the presence of all the markers in the genome, and the determination of genomic distribution of these 5123 ILP markers on the foxtail millet genome revealed physical localization of 4049 markers on the nine chromosomes of foxtail millet with an average marker density of 9.8 markers/Mb (Fig. 1; Table 1). The average marker density was maximum (14.1/Mb) in chromosome 9, followed by chromosome 5 (11.8/Mb), and minimum in chromosome 8 (5.7/Mb). An extensive analysis of chromosome-wise distribution and frequency of these physically mapped ILP markers showed higher frequency of markers mapped on chromosome 9 (831 markers, 20.5%) and minimum on chromosome 8 (230, 5.7%) (Fig. 1; Table 1).

**Table 1.** Summary of chromosomal distribution and average physical density of ILP markers mapped on the nine chromosomes of foxtail millet

Chromosome	Markers mapped (%)	Physical density (markers/Mb)
Chr.1	457 (11.3)	10.9
Chr.2	506 (12.5)	10.3
Chr.3	487 (12)	9.6
Chr.4	325 (8)	8.1
Chr.5	558 (13.8)	11.8
Chr.6	278 (6.9)	7.7
Chr.7	377 (9.3)	10.5
Chr.8	230 (5.7)	5.7
Chr.9	831 (20.5)	14.1
Average		9.8

### 3.2. Functional annotation of ILP

**Figure 2.**
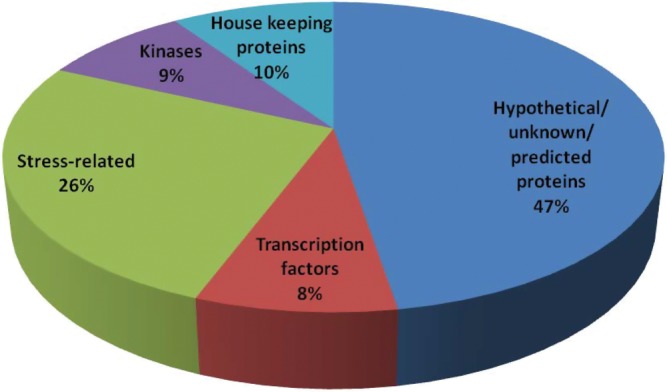
Functional classification of 5123 ILP markers containing ESTs/genes*.* The unique genes were grouped into five functional groups.

BLASTX analyses of the 5123 EST sequences suggested a nearly defined function for ∼71.5% of ILP markers, and 28.5% had no similarities to previously sequenced genes. Based on the function, the ILP markers with defined function (71.5%) were grouped into five major categories (Fig. 2). The largest category (47.4%) contained EST sequences with hypothetical/uncharacterized/putative functions. The second largest category (26.2%) comprised stress-related transcripts. The housekeeping proteins (9.5) ranked third, followed by protein kinases (8.6%) and transcription factors (8.3%) (Fig. 2). Foxtail millet being a potentially abiotic stress-tolerant crop particularly towards drought and salinity, investigating the EST sequences with hypothetical/uncharacterized/putative functions (47.4%) and those which no similarities to previously sequenced genes (28.5%) could provide novel clues on the stress tolerance mechanisms.

### 3.3. Marker validation, cross-genera transferability and genetic basis of sequence length variation

**Figure 3.**
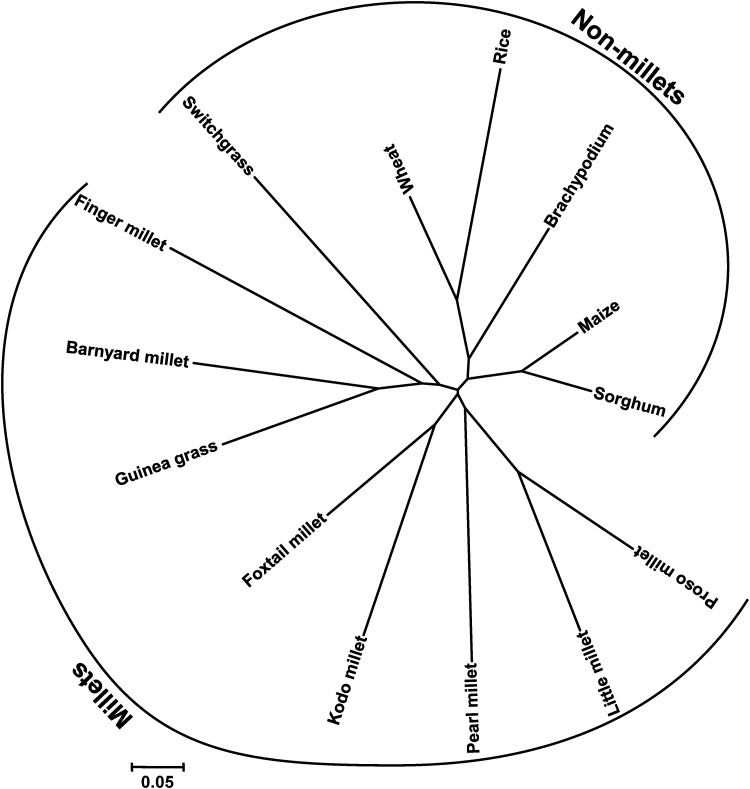
Genetic relationships among millet and non-millet grass species based on 43 foxtail millet ILP markers, using NJoin clustering. Nine millet species including foxtail millet clearly differentiated from the five non-millet grass species and expected genetic relationships among species under study were also apparent.

To amplify introns by PCR, the exon-primed intron-crossing PCR (EPIC-PCR) method was used,^30^ where primers were designed in flanking exons using the PIP database. The advantages of EPIC-PCR are it is fast, reliable, reproducible and convenient, providing ready-to-use and clearly intelligible results. Hence, from the 4049 physically mapped ILP markers, 440 primer pairs flanking the exons were chosen for further analyses based on two criteria, viz. representing the whole genome of foxtail millet and the function of the EST. All the ILP markers were evidenced to produce clear, successful and reproducible amplification in *S. italica* cv. Prasad with 100% amplification potential (Supplementary Table S3). This demonstrates the significance of the developed 4049 ILP markers in expediting foxtail millet genomics and molecular breeding. About 391 (∼90%) of the 440 ILP markers amplified unique single allele, while 49 markers amplified more than one allele/multiple alleles. Thus, a total of 495 alleles were amplified by 440 ILP markers in *S. italica* cv. Prasad (Supplementary Table S3). All the 440 ILP markers have the ability to distinguish the investigated millet and non-millet species into two distinct groups (Fig. 3).

From these validated set of 440 ILP markers, about 100 markers representing the whole foxtail millet genome were chosen to evaluate its polymorphism and molecular diversity potential in a set of eight accessions of *Setaria* including five cultivated and three wild species. About 45 (45%) SiILP markers showed polymorphism and a total of 163 alleles ranging from 1 to 5 alleles were amplified by candidate SiILP markers with an average of 2.15 alleles per marker locus (Supplementary Table S4). The polymorphic potential (∼45%) of ILP markers estimated among foxtail millet cultivated and wild accessions was higher than that reported using the ILP markers derived from foxtail millet genomic sequences.^20^ The higher polymorphic potential of ILP markers is expected because of their development targeting the hypervariable introns, which are under less selective pressure.

**Figure 4.**
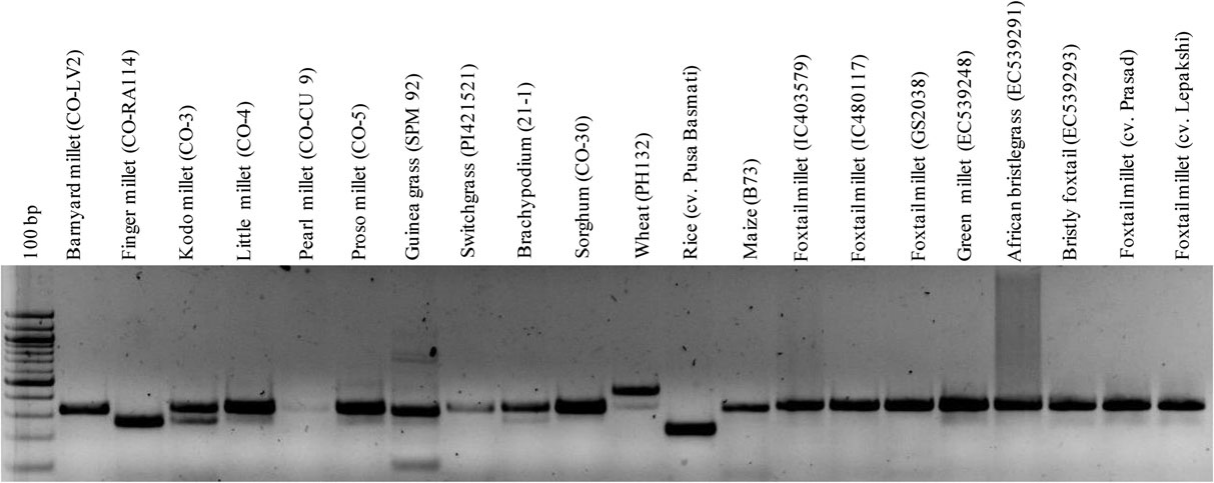
Representative gel showing amplification profiles of one ILP marker SiILP4686 and its fragment-length polymorphism among foxtail millet and related species. The amplicons are resolved in 2% agarose gel along with 100 bp DNA size standard.

In order to investigate the utility of the ILP markers in cross-genera transferability, the 100 validated set of ILP markers were used to amplify the genomic DNA of millet (barnyard millet, finger millet, kodo millet, little millet, pearl millet, proso millet, guinea grass) and non-millet species (switchgrass, sorghum, maize, rice, *Brachypodium*) (Table 2; Fig. 4). Of the 100 SiILP markers assayed, the highest transferability percentage (98%) was observed in proso millet and lowest (59.4%) in wheat, with an average percent transferability of ∼85% (Table 2). Markers which showed a consistent amplification profile in other species were scored as being cross-transferable, thus confirming the utility of developed ILP markers for revealing high cross-genera transferability. To gain further insight into the molecular basis of cross-transferability of ILP markers, the paralogous relationships of 4049 markers orthologous between foxtail millet and rice genes were determined. Three hundred and forty-seven (8.6%) of 4049 orthologous ILP markers were present in paralogous foxtail millet genes, while 80 (2%) were found in paralogous rice genes. It inferred that 3622 ILP markers designed in this study are unique either in the foxtail millet or in the rice genome. Interestingly, the 100 ILP markers showing orthologous relationships between foxtail millet and rice genes selected for cross-transferability study did not show any paralogous relationships within either rice or foxtail millet genome. This confirms the higher cross-transferability potential of ILP markers due to orthologous relationships among species rather than paralogy within species.

**Table 2.** Percent transferability of 100 ILP markers in different millet and non-millet species

No.	Investigated crop	% Transferability
1	Barnyard millet	94.1
2	Finger millet	81.2
3	Kodo millet	74.3
4	Little millet	97.0
5	Pearl millet	96.0
6	Proso millet	98.0
7	Guinea grass	91.1
8	Switchgrass	86.1
9	*Brachypodium*	67.3
10	Sorghum	89.1
11	Wheat	59.4
12	Rice	81.2
13	Maize	88.1
Average		84.8

To examine whether the PCR products were really amplified or homologous to the target genes in millet and non-millet species, we randomly picked up a primer pair, SiILP4686 (*Zea mays* PHD transcription factor), which amplified variant alleles from 214 to 455 bp (Fig. 4). As expected, the sequences of cloned PCR products of the investigated species revealed indels and several point mutations, such as single-base insertions, deletions or translocations; in addition, polymorphism in intron length was observed (data not shown). Overall, multiple sequence alignment has shown that they were homologous to each other and comprised conserved exon regions at two end positions and non-conserved or variable intron region in the middle. Similar kind of observation was also reported in rice,^18^ *Hypericum perforatum*^31^ and foxtail millet.^20^ Further, higher level of transferability of ILPs compared with previously identified markers reflects the conserved nature of exon positions in gene and variability in the non-coding sequences.^18^ Hence, in our study, the high levels of cross-species amplification indicate that the foxtail millet ILP markers could be successfully useful for comparative mapping in millet and non-millet species. Similarly, it was shown that EST-SSR has higher cross-species transferability than genomic SSR markers.^10,32,33^ In addition, all the 100 ILP markers possess ability to distinguish the investigated millet and non-millet species belonging to different genera. The variations in the number of alleles per ILP marker locus in different species studied are possibly dependent upon ploidy level, nature and number of genotype sets in each species used for analysis.

### 3.4. Genetic diversity

**Figure 5.**
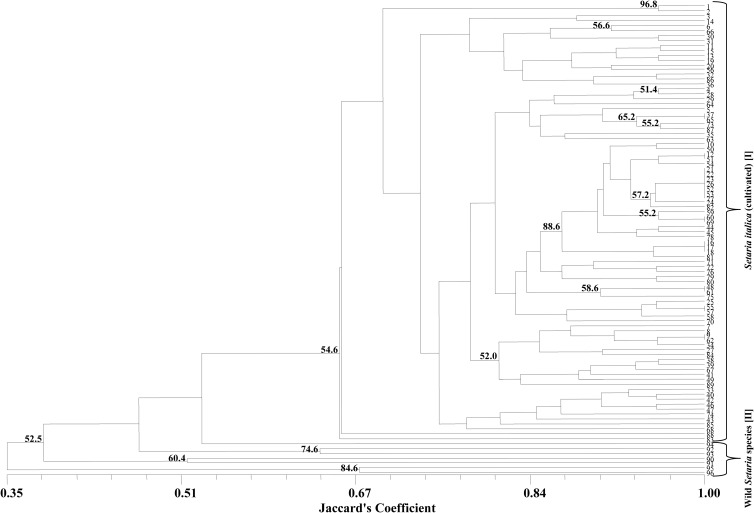
Phylogenetic relationships among cultivated and wild *Setaria* species, using 20 ILP markers. Serial numbers of the accessions correspond to Supplementary Table S1. Numbers at branch points indicate support for *Setaria* species clustered, and values are percent of bootstrap (>50% are indicated) sample that exhibited the cluster.

Besides, the validated physically mapped markers could enable one to discriminate all the 96 cultivated and wild foxtail millet accessions from each other with a level diversity from 0 to 65% (Fig. 5). A core set of 89 cultivated *S. italica* accessions and 7 related wild species were used to decipher the polymorphic potential of 20 ILP markers representing the whole genome of foxtail millet. In total, 59 alleles were identified with an average of about 3 alleles per locus, varying from 2 to 5 (Table 3). This was comparable with a recent study in foxtail millet using ILP markers,^20^ where the average number of alleles per locus reported was 2.6. The polymorphic information content (PIC) values were extended from 0.03 to 0.47 with a mean of 0.20. The observed heterozygosity (*H*_O_) for individual loci ranged from 0.00 to 0.32, with a mean of 0.13 (Table 3). The PIC value was calculated in order to examine the extent of information on diversity that these markers can provide and compare these results with previous published studies. The average PIC value (0.20) reported in this study was lower than that reported for rice (0.45 and 0.44).^18,34^ The probable reason for differences in results might be attributed to the difference in number of genotypes and their genetic background and number of markers used. Nei's average gene diversity (*Nei*) ranged from 0.03 to 0.52, with a mean of 0.22. Among all the loci analysed with fixation index (*F*_IS_), 15 loci were found positive, representing excess of observed homozygotes, whereas 5 loci were negative, demonstrating heterozygotes, with a mean of 0.42 per locus (Table 3). There was no significant correlation observed between PIC and allele number for the 20 markers investigated (data not shown). The phylogenetic tree constructed in this study using ILP markers differentiated 89 cultivated *S. italica* accessions and 7 related wild species from each other and clustered according to their taxonomic classification (Fig. 5). The dendrogram constructed grouped 96 *Setaria* accessions into two distinct clusters, cluster I with 89 accessions comprising cultivated species (foxtail millet, *S. italica*) and cluster II includes the wild *Setaria* species (Fig. 5). Therefore, the ILP markers with high amplification and polymorphic potential distributed over nine chromosomes of foxtail millet genome could be promisingly useful for many large-scale genotyping applications in foxtail millet.

**Table 3.** Summary of genetic diversity estimates of 96 foxtail accessions using 20 ILP markers

Locus name	*N*_A_	*Nei*	*H*o	PIC	*F*_IS_
SiILP1028	3	0.09	0.01	0.09	0.89
SiILP1422	3	0.16	0.10	0.15	0.34
SiILP2672	2	0.31	0.00	0.26	1.00
SiILP709	3	0.52	1.00	0.40	−0.92
SiILP1077	3	0.10	0.01	0.10	0.90
SiILP1496	3	0.50	0.03	0.44	0.94
SiILP4686	3	0.27	0.11	0.25	0.57
SiILP3602	3	0.16	0.17	0.15	−0.02
SiILP431	2	0.05	0.01	0.05	0.80
SiILP996	3	0.08	0.02	0.08	0.74
SiILP1412	4	0.06	0.03	0.06	0.49
SiILP23	4	0.11	0.03	0.11	0.72
SiILP497	3	0.42	0.14	0.37	0.67
SiILP3434	3	0.34	0.32	0.30	0.06
SiILP3864	3	0.14	0.15	0.13	−0.05
SiILP45	5	0.51	0.00	0.47	1.00
SiILP159	3	0.21	0.10	0.20	0.51
SiILP34	2	0.03	0.03	0.03	−0.01
SiILP1365	3	0.07	0.01	0.07	0.85
SiILP9	2	0.26	0.26	0.22	−0.01
Average	3	0.22	0.13	0.20	0.42

*N*_A_, number of alleles; *H*_O_, observed heterozygosity; *Nei*, Nei's average gene diversity; *F*_IS_, fixation index; PIC, polymorphic information content.

### 3.5. *In silico* comparative genome mapping between foxtail millet and other grass species

**Figure 6.**
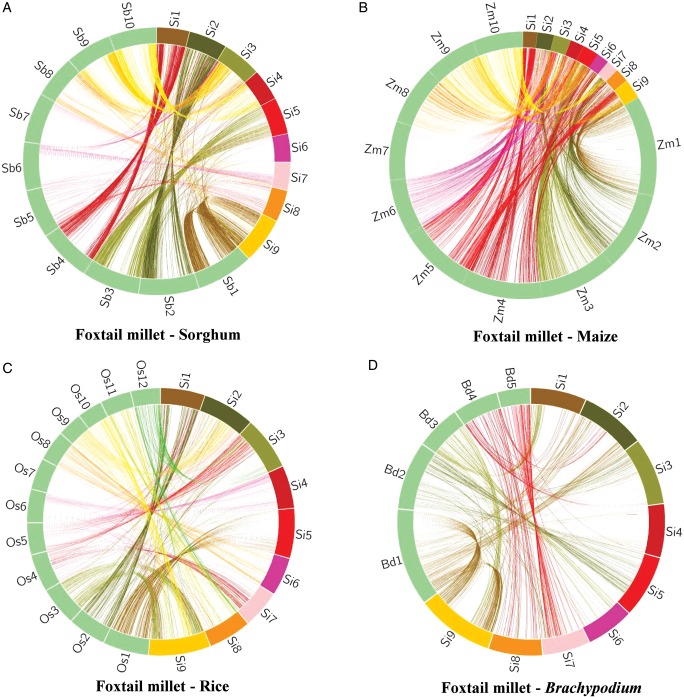
Genome relationships of foxtail millet with other grass species. Comparative mapping between foxtail millet chromosomes with (a) sorghum; (b) maize; (c) rice and (d) *Brachypodium* chromosomes, using 4049 physically mapped foxtail millet ILP markers. Maximum syntenic relationships of foxtail millet chromosomes with sorghum chromosomes based on ILP markers was evident.

In order to substantiate that the ILP marker-based physical map constructed in this study for foxtail millet genome could be useful in comparative genome mapping, the physically mapped 4049 SiILP markers were compared with their physical location on the chromosomes of other related grass genomes, including sorghum, maize, rice and *Brachypodium* (Fig. 6; Table 4). The comparative genome mapping showed considerably significant proportion of sequence-based orthology and syntenic relationship of SiILP markers distributed over nine foxtail millet chromosomes with sorghum (∼50%, 2038), maize (∼46%, 1867), rice (∼21%, 868) and *Brachypodium* (∼21%, 845) chromosomes (Fig. 6; Supplementary Tables S5–S8).

**Table 4.** A summary of ILP marker-based comparative mapping showing maximum syntenic relationships of foxtail millet chromosomes with sorghum, maize, rice and *Brachypodium* chromosomes

Foxtail millet chromosomes	Sorghum chromosomes	Maize chromosomes	Rice chromosomes	*Brachypodium* chromosomes
Chr.1	Chr.4 (221, 92.9%)	Chr.5 (117, 53.9%), Chr.4 (85, 39.2%)	Chr.2 (75, 85.2%)	Chr.3 (88, 84.6%)
Chr.2	Chr.2 (241, 87%)	Chr.7 (143, 58.6%), Chr.2 (65, 26.6%),	Chr.7 (57, 48.3%)	Chr.1 (49, 47.6%)
Chr.3	Chr.9 (158, 61.7%)	Chr.6 (67, 29.8%), Chr.8 (64, 28.4%)	Chr.5 (48, 60.8%)	Chr.2 (59, 58.4%)
Chr.4	Chr.10 (134, 85.4%)	Chr.9 (64, 42.4%), Chr.6 (42, 27.8%)	Chr.6 (74, 83.1%)	Chr.1 (64, 83.1%)
Chr.5	Chr.3 (274, 88.7%)	Chr.3 (146, 50.7%), Chr.8 (101, 35.1%)	Chr.1 (117, 82.4%)	Chr.2 (108, 90%)
Chr.6	Chr.7 (116, 85.9%)	Chr.1 (37, 32.2%), Chr.4 (36, 31.3%)	Chr.8 (42, 84%)	Chr.3 (41, 82%)
Chr.7	Chr.6 (114, 66.7%)	Chr.2 (64, 39.5%), Chr.10 (50, 30.9%)	Chr.7 (51, 64.6%)	Chr.5 (53, 66.3%)
Chr.8	Chr.5 (59, 75.6%)	Chr.4 (34, 44.7%), Chr.2 (25, 32.9%)	Chr.11 (29, 70.7%)	Chr.4 (29, 76.3%)
Chr.9	Chr.1 (380, 91.1%)	Chr.1 (221, 56.8%), Chr.5 (73, 18.8%)	Chr.3 (123, 67.6%)	Chr.1 (130, 75.6%)

#### 3.5.1. Foxtail millet–sorghum synteny

The comparative mapping showed a syntenic relationship of 2038 ILP marker loci distributed over 9 chromosomes of foxtail millet with 2038 genomic regions on 10 chromosomes of sorghum (Fig. 6a; Supplementary Table S5). About 50% syntenic relationship of ILP marker loci between foxtail millet and sorghum chromosomes was observed on an average, with maximum synteny between foxtail millet chromosome 9 and sorghum chromosome 1 (∼91%), followed between foxtail millet chromosome 5 and sorghum chromosome 3 (∼89%) (Fig. 6a; Supplementary Table S5).

#### 3.5.2. Foxtail millet–maize synteny

About 1867 ILP marker loci distributed over nine chromosomes of foxtail millet showed significant matches with 1867 genomic regions of 10 maize chromosomes (Fig. 6b; Supplementary Table S6). Interestingly, each foxtail millet chromosome showed sytenic relationship with two maize chromosomes, thus highlighting the recent whole-genome duplication in maize. All the nine foxtail millet chromosomes showed considerable and higher average frequency (∼46%) of ILP marker-based syntenic relationship with specific maize chromosomes. The physically mapped ILP markers on the foxtail millet chromosome 9 showed maximum synteny (∼57%) with maize chromosome 1 and between foxtail millet chromosome 5 and maize chromosome 3 (∼51%) (Fig. 6b; Supplementary Table S6).

#### 3.5.3. Foxtail millet–rice synteny

The syntenic relationship of 868 ILP marker loci distributed over nine chromosomes of foxtail millet with 868 genomic regions on 12 chromosomes of rice was evidenced (Fig. 6c; Supplementary Table S7). Maximum synteny of ILP marker loci was observed between foxtail millet chromosome 9 and rice chromosome 3 (∼68%) and between foxtail millet chromosome 5 and rice chromosome 1 (∼83%) (Fig. 6c; Supplementary Table S7).

#### 3.5.4. Foxtail millet–*Brachypodium* synteny

The ILP marker-based comparative mapping showed a similar pattern of synteny between foxtail millet and rice. *Brachypodium* chromosomes demonstrated a syntenic relationship with an average frequency of ∼21% (845 marker loci) (Fig. 6d; Supplementary Table S8). The physically mapped ILP markers on the foxtail millet chromosome 9 showed maximum synteny (∼76%) with *Brachypodium* chromosome 1 (Fig. 6d; Supplementary Table S8).

Though there are many reports showing the mapping of microsatellite markers either genetically or physically on orthologous or syntenic chromosomes of different related plant genomes,^7–9,35–38^ this is the first report of comparative mapping using ILP markers. The syntenic relationships showed a higher degree of synteny between foxtail and sorghum genome followed by maize, rice and *Brachypodium*, which is possibly due to their taxonomic relationship, where foxtail millet, sorghum and maize belong to same subfamily Panicoideae, while rice belongs to Ehrhartoideae and *Brachypodium* belongs to Pooideae. The comparative mapping thus demonstrates the decrease of colinearity with increasing phylogenetic distance among plant species. These results are in accordance with the interpretations reported using genomic SSR markers^8^ and genic EST-derived SSR markers^9^ in foxtail millet. This shows the applicability of ILP marker-based comparative genome mapping between foxtail millet and other grass species such as sorghum, maize, rice and *Brachypodium* in translating the sequence information/candidate genes from this diploid crop to other polyploid biofuel grasses. Further, the ILP marker-based comparative mapping between foxtail millet and other grass species could enable transfer of gene-based marker information among these target species and thus would expedite map-based isolation of genes of agronomic importance in foxtail millet.

## 4. Conclusions

To the best of our knowledge, the ILP markers developed in this study are the large-scale novel set of markers in foxtail millet in addition to 98 earlier reported by Gupta *et al*.^20^ The present study identified 5123 ILP markers from 24 828 non-redundant ESTs, of which 4049 markers were physically mapped onto 9 chromosomes of foxtail millet genome. The validation, cross-genera transferability and genetic diversity studies demonstrated expediency of these ILP markers in germplasm characterization, genome relationships in millet and non-millet species and comparative mapping. The newly developed large-scale SiILP markers will be made available to the research community through the FmMDb (http://www.nipgr.res.in/foxtail.html)^10^ and this will promisingly expedite the molecular breeding in foxtail millet and other millets and forage grass species.

## Supplementary Data

Supplementary Data are available at www.dnaresearch.oxfordjournals.org.

## Funding

The authors' work in this area was supported by the core grant of NIPGR. M.M. and G.P. acknowledge the award of Junior Research Fellowship from University Grants Commission, New Delhi.

## Supplementary Material

Supplementary Data
