# Supplementary material for: Development of 5123 Intron-Length Polymorphic Markers for Large-Scale Genotyping Applications in Foxtail Millet
Source: DNA Res. 2013 Oct 1;21(1):41–52. doi: 10.1093/dnares/dst039 (PMC3925393; doi:10.1093/dnares/dst039)
Supplement: Supplementary Data [file supp_21_1_41__index.html]

Development of 5123 Intron-Length Polymorphic Markers for Large-Scale Genotyping Applications in Foxtail Millet — Development of 5123 Intron-Length Polymorphic Markers for Large-Scale Genotyping Applications in Foxtail Millet — Supplementary Data 

# Development of 5123 Intron-Length Polymorphic Markers for Large-Scale Genotyping Applications in Foxtail Millet

## Supplementary Data

Supplementary Data

**Files in this Data Supplement:**

- Supplementary Table 1 - doc file
- Supplementary Table 2 - xls file
- Supplementary Table 3 - xls file
- Supplementary Table 4 - xls file
- Supplementary Table 5 - xls file
- Supplementary Table 6 - xls file
- Supplementary Table 7 - xls file
- Supplementary Table 8 - xls file
