# Supplementary material for: Development of 5123 Intron-Length Polymorphic Markers for Large-Scale Genotyping Applications in Foxtail Millet
Source: DNA Res. 2013 Oct 1;21(1):41–52. doi: 10.1093/dnares/dst039 (PMC3925393; doi:10.1093/dnares/dst039)
Supplement: Supplementary Data [file supp_dst039_dst039supp_table1.doc]

**Supplementary Table S1: Genotypes used in the present study**

| **S.No.** | **Common name** | **Accession no** | **Origin** | **Genera /Species** |
| --- | --- | --- | --- | --- |
| 1 | Foxtail millet | cv.Prasad | India | *Setaria italica* |
| 2 | Foxtail millet | cv. Lepakshi | India | *S. italica* |
| 3 | Foxtail millet | IC-403579 | India | *S. italica* |
| 4 | Foxtail millet | IC-403846 | India | *S. italica* |
| 5 | Foxtail millet | IC-403989 | India | *S. italica* |
| 6 | Foxtail millet | IC-403994 | India | *S. italica* |
| 7 | Foxtail millet | IC-404103 | India | *S. italica* |
| 8 | Foxtail millet | IC-404112 | India | *S. italica* |
| 9 | Foxtail millet | IC-404133 | India | *S. italica* |
| 10 | Foxtail millet | IC-404144 | India | *S. italica* |
| 11 | Foxtail millet | IC-403522 | India | *S. italica* |
| 12 | Foxtail millet | IC-403717 | India | *S. italica* |
| 13 | Foxtail millet | IC-403739 | India | *S. italica* |
| 14 | Foxtail millet | IC-403824 | India | *S. italica* |
| 15 | Foxtail millet | IC-480833 | India | *S. italica* |
| 16 | Foxtail millet | IC-480833B | India | *S. italica* |
| 17 | Foxtail millet | IC-480554 | India | *S. italica* |
| 18 | Foxtail millet | IC-403899A | India | *S. italica* |
| 19 | Foxtail millet | IC-480415 | India | *S. italica* |
| 20 | Foxtail millet | IC-480117 | India | *S. italica* |
| 21 | Foxtail millet | IC-479442 | India | *S. italica* |
| 22 | Foxtail millet | IC-479578 | India | *S. italica* |
| 23 | Foxtail millet | IC-480201 | India | *S. italica* |
| 24 | Foxtail millet | IC-479481 | India | *S. italica* |
| 25 | Foxtail millet | IC-479674 | India | *S. italica* |
| 26 | Foxtail millet | IC-479419 | India | *S. italica* |
| 27 | Foxtail millet | IC-479883 | India | *S. italica* |
| 28 | Foxtail millet | IC-479731 | India | *S. italica* |
| 29 | Foxtail millet | IC-479984 | India | *S. italica* |
| 30 | Foxtail millet | IC-479887 | India | *S. italica* |
| 31 | Foxtail millet | IC-479770 | India | *S. italica* |
| 32 | Foxtail millet | IC-480424 | India | *S. italica* |
| 33 | Foxtail millet | IC-479582 | India | *S. italica* |
| 34 | Foxtail millet | IC-479411 | India | *S. italica* |
| 35 | Foxtail millet | IC-480104 | India | *S. italica* |
| 36 | Foxtail millet | IC-479586 | India | *S. italica* |
| 37 | Foxtail millet | IC-479560 | India | *S. italica* |
| 38 | Foxtail millet | IC-479243 | India | *S. italica* |
| 39 | Foxtail millet | IC-426717 | India | *S. italica* |
| 40 | Foxtail millet | IC-436928 | India | *S. italica* |
| 41 | Foxtail millet | IC-340216 | India | *S. italica* |
| 42 | Foxtail millet | IC-276598 | India | *S. italica* |
| 43 | Foxtail millet | GS450 | India | *S. italica* |
| 44 | Foxtail millet | GS451 | India | *S. italica* |
| 45 | Foxtail millet | GS452 | India | *S. italica* |
| 46 | Foxtail millet | GS460 | USA | *S. italica* |
| 47 | Foxtail millet | GS461 | USA | *S. italica* |
| 48 | Foxtail millet | GS463 | USA | *S. italica* |
| 49 | Foxtail millet | GS464 | USA | *S. italica* |
| 50 | Foxtail millet | GS465 | USA | *S. italica* |
| 51 | Foxtail millet | GS470 | USA | *S. italica* |
| 52 | Foxtail millet | GS473 | USA | *S. italica* |
| 53 | Foxtail millet | GS475 | USA | *S. italica* |
| 54 | Foxtail millet | GS478 | USA | *S. italica* |
| 55 | Foxtail millet | GS479 | USA | *S. italica* |
| 56 | Foxtail millet | GS480 | USA | *S. italica* |
| 57 | Foxtail millet | GS481 | USA | *S. italica* |
| 58 | Foxtail millet | GS482 | USA | *S. italica* |
| 59 | Foxtail millet | GS485 | USA | *S. italica* |
| 60 | Foxtail millet | GS486 | USA | *S. italica* |
| 61 | Foxtail millet | GS499 | USA | *S. italica* |
| 62 | Foxtail millet | GS500 | USA | *S. italica* |
| 63 | Foxtail millet | GS502 | USA | *S. italica* |
| 64 | Foxtail millet | GS503 | USA | *S. italica* |
| 65 | Foxtail millet | GS505 | USA | *S. italica* |
| 66 | Foxtail millet | GS507 | USA | *S. italica* |
| 67 | Foxtail millet | GS511 | USA | *S. italica* |
| 68 | Foxtail millet | GS455 | US/AFRICA | *S. italica* |
| 69 | Foxtail millet | GS488 | China | *S. italica* |
| 70 | Foxtail millet | GS489 | China | *S. italica* |
| 71 | Foxtail millet | GS490 | China | *S. italica* |
| 72 | Foxtail millet | GS491 | China | *S. italica* |
| 73 | Foxtail millet | GS492 | China | *S. italica* |
| 74 | Foxtail millet | GS1641 | China | *S. italica* |
| 75 | Foxtail millet | GS2037 | China | *S. italica* |
| 76 | Foxtail millet | GS2038 | China | *S. italica* |
| 77 | Foxtail millet | GS2039 | China | *S. italica* |
| 78 | Foxtail millet | GS494 | Kenya | *S. italica* |
| 79 | Foxtail millet | GS495 | Kenya | *S. italica* |
| 80 | Foxtail millet | GS496 | Kenya | *S. italica* |
| 81 | Foxtail millet | GS497 | Kenya | *S. italica* |
| 82 | Foxtail millet | GS498 | Kenya | *S. italica* |
| 83 | Foxtail millet | GS1926 | Bangladesh | *S. italica* |
| 84 | Foxtail millet | GS1928 | Bangladesh | *S. italica* |
| 85 | Foxtail millet | GS1643 | USSR | *S. italica* |
| 86 | Foxtail millet | GS1646 | USSR | *S. italica* |
| 87 | Foxtail millet | GS493 | Turkey | *S. italica* |
| 88 | Foxtail millet | GS1636 | Pakistan | *S. italica* |
| 89 | Foxtail millet | GS454 | Ethiopia | *S. italica* |
| 90 | Green millet | EC539248 | India | *S. viridis* |
| 91 | Green millet | EC539251 | India | *S. viridis* |
| 92 | African bristlegrass | EC539290 | India | *S. sphacelata* |
| 93 | African bristlegrass | EC539291 | India | *S. sphacelata* |
| 94 | Bristly foxtail | EC539293 | India | *S. verticillata* |
| 95 | Bristly foxtail | EC539297 | India | *S. verticillata* |
| 96 | Bristly foxtail | EC539300 | India | *S. verticillata* |
| 97 | Barnyard millet | CO-LV2 | - | *Echinochloa frumentacea* |
| 98 | Finger millet | CO-RA114 | - | *Eleusine coracana* |
| 99 | Kodo millet | C0-3 | - | *Paspalum scrobiculatum* |
| 100 | Little millet | C0-4 | - | *Panicum sumatrense* |
| 101 | Pearl millet | CO-CU 9 | - | *Pennisetum glaucum* |
| 102 | Proso millet | CO-5 | - | *Panicum miliaceum* |
| 103 | Switchgrass | PI421521 | - | *Panicum virgatum* |
| 104 | Guinea grass | SPM92 | - | *Panicum maximum* |
| 105 | Sorghum | CO30 | - | *Sorghum bicolor* |
| 106 | Wheat | PH132 | - | *Triticum aestivum* |
| 107 | Rice | cv. Pusa Basmati | - | *Oryza sativa* |
| 108 | Maize | B73 | - | *Zea mays* |
| 109 | Brachypodium | BD21-1 | - | *Brachypodium distachyon* |
